# Enhancing Fetal Anomaly Detection in Ultrasonography Images: A Review of Machine Learning-Based Approaches

**DOI:** 10.3390/biomimetics8070519

**Published:** 2023-11-02

**Authors:** Ramin Yousefpour Shahrivar, Fatemeh Karami, Ebrahim Karami

**Affiliations:** 1 Department of Biology, College of Convergent Sciences and Technologies, Science and Research Branch, Islamic Azad University, Tehran, 14515-775, Iran; 2 Department of Medical Genetics, Applied Biophotonics Research Center, Science and Research Branch, Islamic Azad University, Tehran, 14515-775, Iran; 3 Department of Engineering and Applied Sciences, Memorial University of Newfoundland, St. John’s, NL A1B 3X5, Canada

**Keywords:** fetal anomaly, prenatal diagnosis, machine learning, deep learning, ultrasonography imaging

## Abstract

Fetal development is a critical phase in prenatal care, demanding the timely identification of anomalies in ultrasound images to safeguard the well-being of both the unborn child and the mother. Medical imaging has played a pivotal role in detecting fetal abnormalities and malformations. However, despite significant advances in ultrasound technology, the accurate identification of irregularities in prenatal images continues to pose considerable challenges, often necessitating substantial time and expertise from medical professionals. In this review, we go through recent developments in machine learning (ML) methods applied to fetal ultrasound images. Specifically, we focus on a range of ML algorithms employed in the context of fetal ultrasound, encompassing tasks such as image classification, object recognition, and segmentation. We highlight how these innovative approaches can enhance ultrasound-based fetal anomaly detection and provide insights for future research and clinical implementations. Furthermore, we emphasize the need for further research in this domain where future investigations can contribute to more effective ultrasound-based fetal anomaly detection.

## 1. Introduction

**Figure 1:**
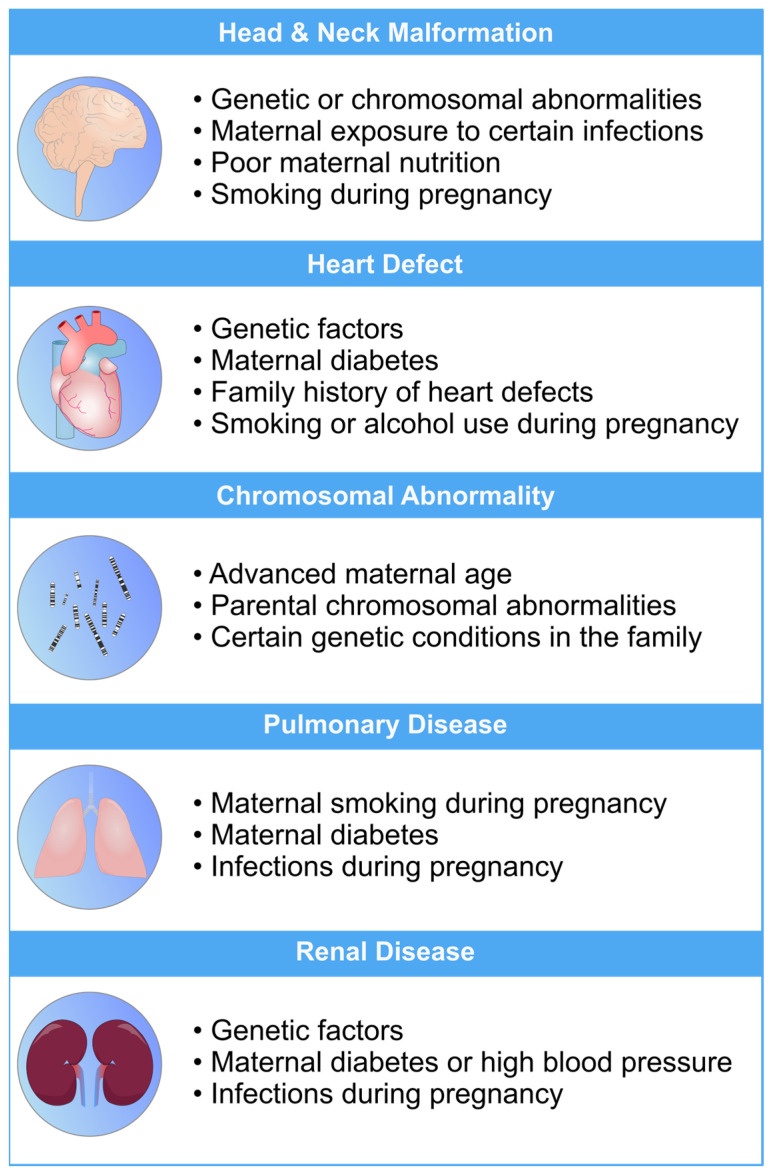
An overview of the most common risk factors associated with fetal abnormalities of the heart, brain, lung, and kidneys. These risk factors can have a profo bcb4und impact on the health and well-being of newborns. Limiting the exposure to these risk factors can mitigate the risk of fetal defects [26,27,28,29,30].

**Figure 2:**
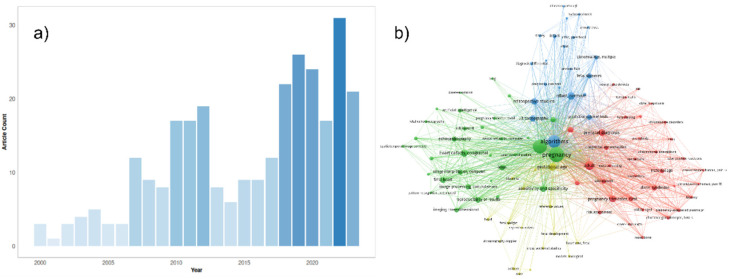
(**a**) An overview of the number of original papers published on PubMed yearly from 2000 to 2023 in this matter. The following PubMed query was used for the generation of this figure: (“Machine Learning” OR “Artificial Intelligence” OR “Machine Learning”[Mesh] OR “Unsupervised Machine Learning”[Mesh] OR “Supervised Machine Learning”[Mesh] OR “Artificial Intelligence”[Mesh] OR “Algorithms”[Mesh] OR “Deep Learning” OR “Algorithm”) AND (“Ultrasound Images” OR “Ultrasonography”[Mesh] OR “Ultrasonography, Prenatal”[Mesh] OR “Echocardiogram” OR “Neurosonography” OR “Echocardiography” OR “Ultrasound”) AND (“Embryonic and Fetal Development”[Mesh] OR “Fetal” OR “Fetus” OR “Fetus”[Mesh] OR “Prenatal”) AND (“abnormalities” OR “anomalies” OR “defects” OR “malformation”) NOT Review[Publication Type]. (**b**) Network representation of the most common keywords in the literature using the same PubMed query results. The three most common keywords in the network are pregnancy, ultrasonography, and algorithms. This network was generated by the authors, using the VOSviewer software version 1.6.19.

Fetal development is a critical phase in human growth, in which any abnormality can lead to significant health complications. The subjectivity and inaccuracies of medical sonographers and technicians in interpreting ultrasonography images often result in misdiagnoses [1,2,3]. Fetal anomalies can be defined as structural abnormalities in prenatal development that manifest in several critical anatomical sites, such as the fetal heart, central nervous system (CNS), lungs, and kidneys (Table 1) [4,5]. These anomalies can arise during various stages of pregnancy and can be caused by different genetics and environmental factors, or a combination of both, which are called multifactorial disorders (Figure 1) [6,7]. Ultrasound and genetic testing are two examples of prenatal screening and diagnostic tools that can help find these abnormalities at an earlier gestational age. Fetal abnormalities can have varying degrees of influence on a child’s health, from those that are easily treatable to those that result in the child’s death either during pregnancy or shortly after birth [8]. The occurrence of fetal anomalies differs across different populations. Structural anomalies in fetuses can be detected in approximately 3% of all pregnancies [9]. Ultrasound (US) is still the most commonly used method to safely screen for fetal anomalies during pregnancy, but it is mainly dependent on sonographer expertise and, therefore, is error-prone. In addition, US images sometimes lack high quality and discrete edges that can lead to inaccurate diagnosis [10,11]. Fetal development is crucial and complex, and abnormalities will significantly impact the children’s and sometimes the maternal health [12]. In this regard, the ever-increasing progress in the field of computer science has produced a wide variety of methods, such as machine learning (ML), deep learning (DL), and neural networks (NN), that are specific techniques within the broader field of artificial intelligence (AI) and have gained notable popularity in the medical field [13,14,15,16,17,18]. These methods include, but are not limited to, image classification, segmentation, detection of specific objects within images, and regression analysis. Consequently, numerous studies have been carried out on developing DL- and ML-based models for the accurate recognition of various types of prenatal abnormalities, including heart defects, CNS malformations, respiratory diseases, and renal anomalies in the context of chromosomal disorders or in the isolated forms. Here, we present a review of the recent state-of-the-art ML-based models for the detection of fetal anomalies. We have searched popular databases such as PubMed, Google Scholar, and Web of Science, and included papers published in high-quartile and impact-factor journals to review the current state of AI in this matter (Figure 2). First, we will give an overview of different ML- and DL-based methods. Second, we will discuss common types of fetal anomalies and the performance of the models that have been employed. Finally, we will discuss some of the challenges that researchers face in this field.

**Table 1:** An overview of various fetal structural anomalies categorized into distinct groups, each associated with specific clinical conditions potentially affecting prenatal development.

Type of Anomaly	Disorders	Refs
Neural Tube Defects (NTDs)	Spina Bifida, Anencephaly	[19]
Heart Defects	Ventricular Septal Defect (VSD), Tetralogy of Fallot	[20]
Gastrointestinal Anomalies	Esophageal Atresia, Anal Atresia, Gastroschisis	[21]
Limb Anomalies	Polydactyly, Syndactyly, Amelia	[21]
Craniofacial Anomalies	Cleft Lip and Palate, Microcephaly	[22]
Genitourinary Anomalies	Hydronephrosis, Renal Agenesis	[23]
Respiratory Anomalies	Congenital Diaphragmatic Hernia, Pulmonary Hypoplasia	[24]
Chromosomal Anomalies	Down Syndrome, Edwards Syndrome, Patau Syndrome	[25]

## 2. Methods in Machine Learning for Fetal Anomaly Detection

**Figure 3:**
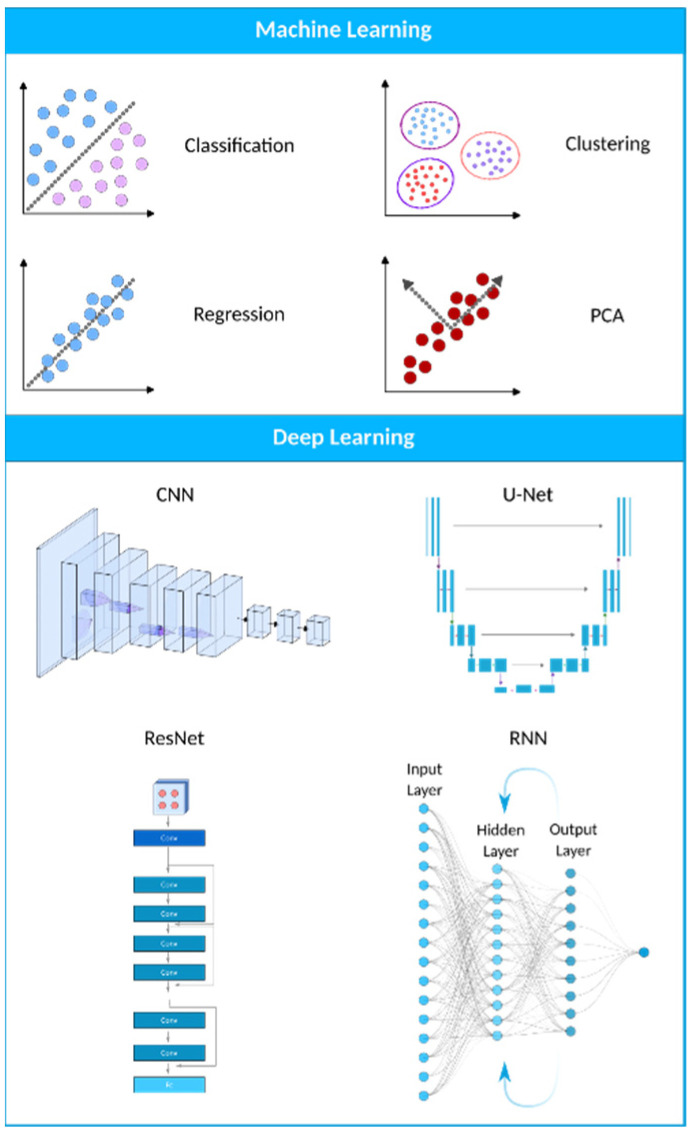
A visual representation of the AI landscape with three primary subsections: artificial intelligence, machine learning, and deep learning. The figure highlights four deep learning models (CNN, U-Net, ResNet, and RNN) and four machine learning algorithms (classification, clustering, PCA, and regression) as key components within these domains.

Machine learning (ML) is a computational technique originated from the field of computer science. In recent years, ML has been extensively used in various fields, such as medical image analysis, and has provided many valuable methods and approaches for more accurate and specific diagnoses. The field of medical image analysis is rapidly evolving, and new models and techniques are constantly emerging (Figure 3). One of the more widely used techniques in this field is deep learning (DL). A recent study has evaluated the practicality of DL-based models within clinics. They have found that AI-driven technologies can significantly help sonographers by performing disruptive tasks automatically, thus allowing technicians to focus mainly on interpreting images [31]. AI-based tools have great potential to lead to a paradigm shift in how we practice medicine. Many researchers have now constructed ML- and DL-based models to use in applications ranging from evaluating gestational age [32] to the simultaneous anomaly detection of fetal organs, which will be discussed in more detail in the following sections.

### 2.1. Deep Learning

The structure and operation of a single neuron directly influenced the biomimetic hypothesis that gave rise to DL. The brain comprises interconnected neurons that handle information and learn from encounters, strengthening connections between neurons that activate simultaneously. Similarly, DL-based models consist of numerous layers of interconnected artificial neurons that imitate this arrangement. Information is processed through these layers of neural connections, with each neuron assigning importance (weights) to inputs and transmitting results to linked nodes. The model learns by fine-tuning connection weights through backpropagation to enhance its capacity to identify patterns, much like neural pathways form in the brain through learning. DL- and ML-based models, in general, develop complex data representations without being explicitly programmed, much like the brain develops cognitive abilities [6,33,34].

ML and DL models can efficiently analyze US images to identify abnormalities and anomalies in fetuses. Using each model has its own advantages and disadvantages (Table 2). ML systems can learn to detect issues like physical defects, growth restrictions, and cardiac anomalies by training algorithms on labeled datasets of normal and abnormal fetal scans. This ability can help obstetricians and radiologists screen for problems and intervene early to improve fetal outcomes. Convolutional neural networks (CNNs) are commonly used for the automated analysis of US images. These algorithms can segment, classify, and quantify anatomical structures to detect anomalies. Other approaches, like generative adversarial networks (GANs), can synthesize fake but normal US images to compare with actual scans [35,36].

#### 2.1.1. Convolutional Neural Networks (CNNs)

CNNs are the most widely utilized deep learning model, and they have had the most success in medical image processing thus far. They have been mostly used for tasks like abnormality detection, organ segmentation, and disease classification. CNNs are becoming more popular because, unlike traditional machine learning algorithms like KNN, SVM, logistic regression, etc., they do not need feature engineering. Due to their excellent performance in medical imaging and their ability to be parallelized with GPUs, CNNs have recently seen widespread adoption within the medical imaging research community [48]. A CNN consists of convolution and pooling layers. Convolution extracts image features by applying small kernels to input pixels, producing feature maps. These maps are passed through activation functions and then downsized by pooling layers, often using max pooling. Multiple convolution and pooling steps create a hierarchy of features. The data are then transformed into a 1D array for classification. CNNs capture image patterns efficiently, making them useful for tasks such as recognizing edges or shapes [37,38,39,49].

**Figure 4:**
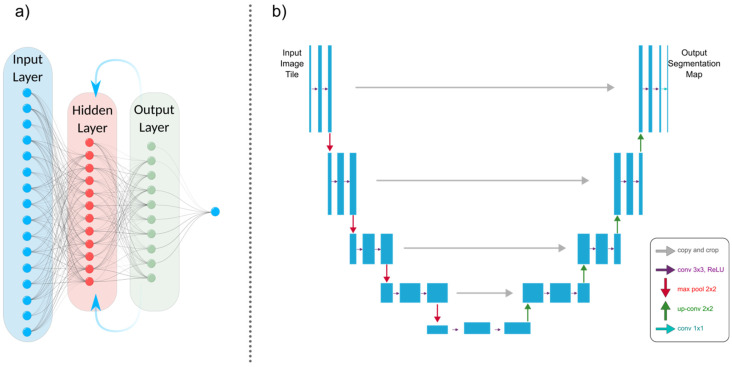
(**a**) An architectural illustration of a recurrent neural network (RNN) demonstrating its essential components. The recurrent connection is indicated by an arrow pointing from the output layer back to the hidden layer. This fundamental connection allows RNNs to sustain a memory of prior inputs and computations. (**b**) A U-Net model’s architectural representation.

U-Net, a subclass of CNN, has gained significant popularity in the medical imaging community for image segmentation tasks due to its effectiveness and efficiency. It was first introduced in 2015 as a novel method for biomedical image segmentation by Ronneberger et al. [50]. The U-Net architecture is named after its U-shaped design, which consists of an encoder path and a corresponding decoder path (Figure 4b). The encoder path progressively reduces the spatial dimensions of the input image while simultaneously extracting high-level features via convolutional and pooling layers. The decoder path then upsamples the feature maps to restore their original spatial resolution, using skip connections to combine low-level and high-level features for precise segmentation [46,50,51]. U-Nets are renowned for their ability to capture fine-grained details and local context, which makes them suitable for biomedical image segmentation, cell detection, and organ localization. Due to their ability to manage limited labeled data and generate accurate segmentation results, they have gained popularity in medical image analysis.

#### 2.1.2. Generative Adversarial Networks (GANs)

**Figure 5:**
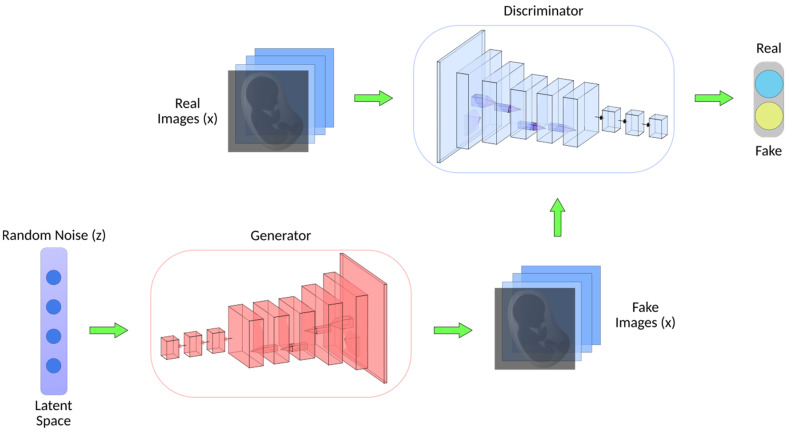
Generative adversarial network (GAN) architecture. GANs consist of two key components. The generator transforms random noise (z) into synthetic/fake images (x), aiming to create realistic images. Simultaneously, the discriminator, which has been trained on real images from a dataset, classifies images as real or fake.

GANs have shown promise in medical image synthesis, augmentation, and translation. They can generate realistic medical images, which can be used for data augmentation, rare disease simulation, and anomaly detection. A GAN is a novel unsupervised learning network that was introduced by Goodfellow et al. in 2014 [52]. This unique neural network architecture involves training two networks at the same time, one for image creation and the other for discriminating between actual and artificially generated images (Figure 5) [53,54]. The critical difference is that CNNs are discriminative models for supervised learning tasks, while GANs are generative models for unsupervised learning problems. A standard GAN has two networks: the generator and the discriminator. The generator aims to produce realistic synthetic data, while the discriminator tries to differentiate between actual and generated data. During training, both networks engage in a two-player minimax game where the generator attempts to deceive the discriminator and the discriminator tries to classify actual and generated samples correctly. One significant advantage is that GANs allow for effective anomaly detection even when training data for abnormal cases are limited [55,56]. This is especially true for studies where large image datasets are not available, such as fetal echocardiograms. The generator learns to produce high-fidelity synthetic images that mimic the distribution of normal cases. Meanwhile, the discriminator learns the patterns of normal anatomy. During testing, real images containing abnormalities would be expected to be classified by the discriminator as fake, allowing for anomaly detection [57]. Additionally, GANs provide continuous learning; as more real fetal image data are collected over time, the networks can be further tuned to improve analysis performance. This is particularly advantageous for the analysis of fetal heart images because the shape of the fetal four-chamber heart (FCH) changes substantially based on the specific gestational week that the fetus is in. As new data from different gestational weeks become available, the GANs can adapt and improve their analysis performance by adjusting their learned representations of the fetal four-chamber heart for different developmental stages [58].

#### 2.1.3. Recurrent Neural Networks (RNNs)

RNNs are a class of neural networks used for processing sequential information, such as time-series analysis or 3D medical image analysis (Figure 4a). They can capture temporal dependencies and have been applied to tasks like cardiac motion analysis, video-based medical diagnosis, and longitudinal disease progression modeling. The most well-known variety of RNNs are LSTM (long short-term memory) networks, a subclass of RNNs. Due to their ability to effectively process sequential data, they are beneficial for medical image analysis tasks. The loss of spatial information is problematic for medical image segmentation when using a typical LSTM network since the inputs must be vectorized [41]. A potential solution is to use a convolutional LSTM, in which the multiplication of vectors is replaced with a convolutional operation [40,42].

## 3. Applications of Machine Learning in Fetal Anomaly Detection

**Figure 6:**
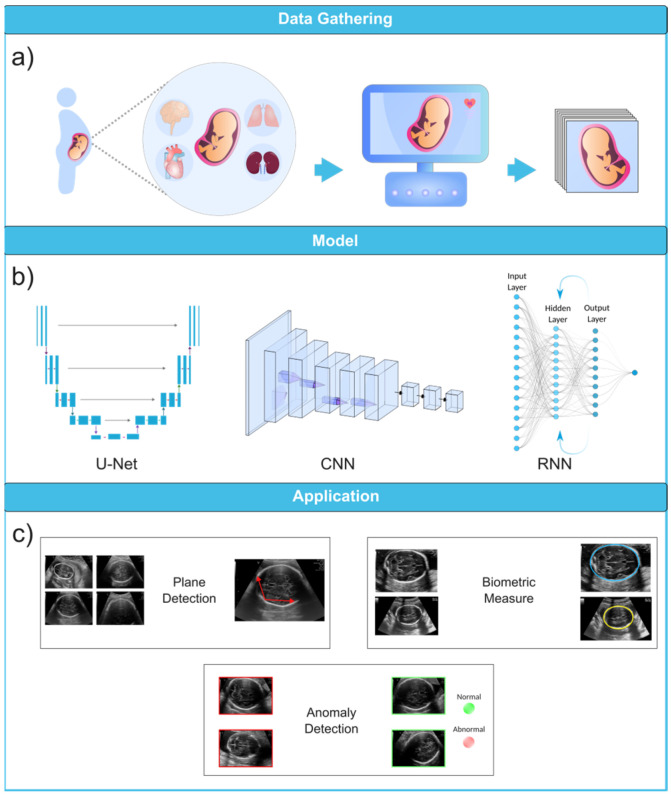
Ultrasound image analysis pipeline. (**a**) In this initial phase, ultrasound imaging is performed on a pregnant woman to identify potential fetal organ abnormalities. (**b**) This section presents a variety of deep learning models designed for different ultrasound image analysis task, such as CNN, U-Net, and RNN. (**c**) This section demonstrates the wide-ranging applications facilitated by deep learning models, including biometric measurements (e.g., head circumference), standard plane identification, and detection of fetal anomalies. Ultrasound images were obtained from the following dataset on the Kaggle (https://www.kaggle.com/datasets/rahimalargo/fetalultrasoundbrain, accessed on 1 August 2023).

To fully appreciate the role of machine learning in the diagnosis of fetal abnormalities, it is necessary to first become familiar with the standard imaging technique that serves as the foundation of this diagnostic procedure. In comparison to computed tomography (CT) and magnetic resonance imaging (MRI), US imaging is the preferable method since it allows for real-time, cost-effective prenatal examination without the use of ionizing radiation. The standard procedure for fetal anomaly detection is typically a multi-step process, starting with the identification and interpretation of the sonographic images (Figure 6a). The initial scans are obtained in the first trimester, followed by a detailed anatomic survey in the second trimester. This survey involves the examination of multiple fetal organ systems and structures like the heart, brain, lungs, and kidneys, among others. Following this, the images are analyzed, pre-processed for any potential noise and errors, and finally fed into ML-based models for the detection of abnormalities or deviations from the normal developmental patterns (Figure 6b,c). ML can significantly streamline this process by automating the initial analysis and potentially identifying abnormalities with greater accuracy and speed than traditional manual interpretation. This section will explore how US imaging works, its advantages, and its ability to capture standard views of fetal structures throughout pregnancy. US is an essential screening and diagnostic technique during all three trimesters of pregnancy, allowing for dynamic viewing of the whole fetus.

### 3.1. Ultrasound Imaging

US imaging provides a real-time, low-cost prenatal evaluation with the additional advantages of being radiation-free and noninvasive in comparison to CT and MRI [59]. During a US exam, a transducer probe is placed against the mother’s abdomen and moved to visualize fetal structures. The probe transmits high-frequency sound waves, which are reflected to produce two-dimensional grayscale images representing tissue planes. The US machine calculates the time interval between transmitted and reflected waves to localize anatomical structures. Repeated pulses and reflections generate real-time visualization of the fetus. The US can capture standard views such as the four-chamber heart, profile, lips, brain, spine, and extremities [60,61,62]. Fetal standard planes in US imaging refer to specific anatomical views to assess fetal development. They provide a standardized orientation for evaluating different structures and measurements in the fetus, aiding in diagnosing potential abnormalities or monitoring the growth and well-being of the developing baby during pregnancy. Thus, the automatic recognition of standard planes in fetal US images is an effective method for diagnosing fetal anomalies. According to the International Society of Ultrasound in Obstetrics and Gynecology (ISUOG) guidelines, there are several types of fetal standard planes (Table 3) [63,64,65].

**Figure 7:**
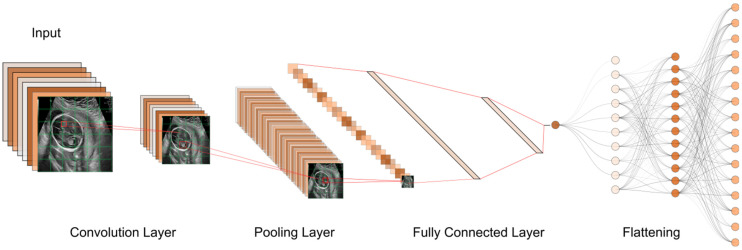
The typical workflow of a CNN for ultrasound image analysis. **Convolution Layer**: This displays the initial layer where input images are processed using convolution operations to extract features. **Pooling Layer**: This illustrates the subsequent layer where pooling operations (e.g., max-pooling) are applied to reduce spatial dimensions and retain important information. **Fully Connected Layer**: This shows the layer responsible for connecting the extracted features to make classification decisions or predictions. **Flattening**: This represents the process of converting the output from the previous layers into a one-dimensional vector for further processing.

Until now, numerous studies have been conducted to find the best models and approaches for reliable US image and video segmentation [66,67,68,69]. The evaluation of fetal health is the most common application of ultrasound technology. In particular, ultrasound is used to monitor the development of a fetus and detect any abnormalities early on. Placenta anomalies, growth restrictions, and structural defects all fall into this category. Due to their improved pattern recognition skills, DL models such as CNN have proven to be effective in the detection of abnormalities (Figure 7). In this context, continuing research on developing novel DL-based image recognition models has the potential to dramatically improve the predicted accuracy of US image segmentation. Table 4 showcases some of the properties of popular DL-based models that are currently being utilized in the medical image analysis field.

Recent advancements in this field have shown great potential for extracting nuanced features from complex fetal US imaging data, which we will discuss in the following sections. Ultimately, integrating DL-based models with the clinical workflow provides automated or semi-automated [76] reliable approaches to efficiently analyzing the nuanced characteristics of individual US scans, thus equipping healthcare providers with a more comprehensive set of tools for fetal health evaluation. The US examination is divided into trimesters to correspond with the three distinct phases of pregnancy, each lasting approximately three months, in order to provide a structured approach for monitoring fetal development and evaluating the health of both mother and child at key points during gestation.

#### 3.1.1. First Trimester

First-trimester US imaging is typically performed between 11 and 13 weeks of gestation [77]. Its primary uses are to confirm pregnancy viability, determine gestational age, evaluate multiple gestations, and screen for significant fetal anomalies such as neural tube defects, abdominal wall defects, cardiac anomalies, nuchal translucency (NT), and some significant fetal brain abnormalities [78,79]. An abnormal NT measurement (≥3.5 mm (>p99)) during the first-trimester US can strongly predict the risk of chromosomal abnormalities and even congenital heart defects [80,81,82].

#### 3.1.2. Second Trimester

Second-trimester US imaging is commonly performed between 18 and 22 weeks of gestation. The primary aim is a detailed anatomical survey to evaluate fetal growth and full screening for structural abnormalities and placental growth and status. The fetal anatomy scan assesses the brain, face, spine, heart, lungs, abdomen, kidneys, and extremities [83]. The second-trimester US has high detection rates for major fetal anomalies if performed by a qualified expert. The appropriateness criteria provide screening recommendations for fetuses in the second and third trimesters with varying risk levels (Table 5) [84]. These guidelines are essential for healthcare providers to ensure proper prenatal care and informed decision making for expectant mothers and their developing fetuses.

#### 3.1.3. Third Trimester

Third-trimester US imaging is often performed around 28–32 weeks of gestation to re-confirm fetal growth and position, screen for anomalies that may have developed since the prior scan, and make further assessments on the placental location and growth. It was found that fetal anomalies can be discovered in 1/300 pregnancies during routine third-trimester ultrasounds [85]. While US is valuable for prenatal screening, it does have limitations. The imaging quality can be impaired by the maternal body environment, fetal position, shadowing from bones, and low amniotic fluid volume [86,87]. Interpretation requires extensive training and is subject to human error. A computerized analysis of US images using ML offers the potential to overcome some human limitations. ML methods aim to improve screening accuracy and standardize interpretation by applying AI to analyze US data. These models can be trained to identify anomalies in poor-quality scans and detect subtle or complex patterns that may be missed by the technicians. However, further research is still needed to fully integrate ML into clinics and medical workflows.

### 3.2. Diagnosis of Fetal Abnormalities

#### 3.2.1. Congenital Heart Diseases

Congenital heart diseases (CHDs) are classified as common and severe congenital malformations in fetuses, occurring in approximately 6 to 13 out of every 1000 cases [88]. Although, CHDs may have no prenatal symptoms, they may result in significant morbidities, and even death, later in life. Since heart defects are the most common fetal anomalies among fetuses, research interest in this matter is consequently higher than other types of defects. Evaluating the cardiac function of a fetus is challenging due to the factors such as the fetus’s constant movement, rapid heart rate, small size, limited access, and insufficient expertise in fetal echocardiography among some sonographers, which makes the identification of complex abnormal heart structures difficult and prone to errors [89,90,91]. Fetal echocardiography was introduced about 25 years ago and now needs to incorporate advanced technologies.

The inability to identify CHD during prenatal screening is more strongly influenced by a deficiency in adaptation skills during the performance of the SAS test than by situational variables like body mass index or fetal position. The cardiac images exhibited a considerably higher frequency of insufficient quality in undiscovered instances as compared to identified ones. In spite of the satisfactory image quality, CHD was undetected in 31% of instances. Furthermore, it is worth noting that in 20% of instances when CHD went undiscovered, the condition was not visually apparent despite the presence of high-quality images [92]. This study illustrates the significance and necessity of ML approaches as tools that can successfully reduce the number of undetected CHD cases and enhance the accuracy of prenatal diagnosis.

Echocardiography, a specialized US technique, remains the primary and essential method for early detection of fetal cardiac abnormalities and mortality risk, aimed at identifying congenital heart defects before birth. It is extensively employed during pregnancy, and the obtained images can be used to train DL models like CNN to automate and enhance the identification of abnormalities [93]. An echocardiogram consists of a detailed US test of the fetal heart, performed prenatally; utilizing AI for analyzing echocardiograms holds promise in advancing prenatal diagnosis and improving heart defect screening [94]. In this context, Gong et al. conducted a study wherein they developed an innovative GAN model. Integrating the DANomaly and GACNN (generative adversarial CNN) neural network architectures resulted in the creation of this model. The objective of this study was to train the model using extracted features derived from FCH images obtained from echocardiogram video slices. Moreover, they used an extension of the original GAN model called the Wasserstein generative adversarial network with gradient penalty (WGAN-GP) to extract features from fetal FCH images. They eventually developed a novel DGACNN, intending to identify CHD by combining the GAN discriminator architecture with additional CNN layers. According to the study, the DGACNN model demonstrated an 85% recognition accuracy in detecting fetal congenital heart disease (FHD), surpassing other advanced networks by 1% to 20%. Compared to expert cardiologists in FHD recognition, the proposed network achieved a remarkable 84% accuracy in the test set [95].

While GANs have demonstrated their effectiveness in anomaly detection and generative modeling, it is possible to enhance their analytical performance for intricate tasks like fetal echocardiography assessment by training an ensemble of multiple neural networks and integrating their predictions. The use of an ensemble of neural networks involves the integration of different neural networks in order to address certain machine-learning objectives. The key concept is that an ensemble of multiple neural networks would typically exhibit greater performance compared to any individual network. In this regard, Arnaout et al. trained an ensemble of neural networks to differentiate normal from CHD cases with respect to the guideline-recommended cardiac views. They used 107,823 images from 1326 echocardiograms and ultrasound images of fetuses between 18 and 24 weeks of gestation. A CNN view classifier was used to train a model capable of identifying the five screening views in fetal ultrasounds. Any image that did not correspond to one of the five views specified by guidelines was classified as ‘non-target’, such as the head, foot, or placenta. The results indicated great performance with an area under the curve (AUC) of 0.99 [96].

The four-chamber view facilitates the assessment of cardiac chamber size and the septum. In contrast, the left ventricular outflow tract view offers a visualization of the aortic valve and root. The right ventricular outflow tract view provides insight into the pulmonary valve and artery, and the three-vessel view confirms normal anatomy by showcasing the pulmonary artery, aorta, and superior vena cava. Additionally, the arch view scrutinizes the transverse aortic arch and branching vessels. During routine obstetric US screenings, these five standard views—the four-chamber, left ventricular outflow, right ventricular outflow, three-vessel, and arch views—give a full view of the fetal heart and major blood vessels (Table 6). This inclusive approach allows for detecting various significant congenital heart conditions before birth.

Emphasizing the importance of the four-chamber views, we can delve into a study by Zhou et al. [97]. They introduced a category attention network aimed at simultaneous image segmentation for the four-chamber view. They modified the SOLOv2 model for object instance segmentation. However, SOLOv2 encounters a potential misclassification issue with grids within divisions containing pixels from different instance categories. This discrepancy arises because the category score of a grid might erroneously surpass that of surrounding grids, which affects the final quality of instance segmentation. Certain image portions would become intertwined, leading to challenges in accurate object classification. To address this, the researchers integrated a “category attention module” (CAM) into SOLOv2, creating CA-ISNet. The CAM analyzes various image sections, aiding in accurately determining object categories. The proposed CA-ISNet model underwent training using a dataset of 319 images encompassing the four cardiac chambers of the fetuses. The functionality of this model relies on three distinct branches:

1. Category Branch, responsible for assigning each instance to an appropriate cardiac chamber by predicting the semantic category of the instance.
2. Mask Branch, segmenting the heart chambers within the images.
3. Category Attention Branch. This component learns the category-related information of instances to rectify any inaccurate classifications made by the category branch.

The results demonstrated an average precision rate of 45.64%, with a DICE range of 0.7470 to 0.8199. DICE is an average value of two other measurements, which are precision and recall rate, and it gives us an overall performance rate for models.

Concerning the simultaneous segmentation framework, another study was conducted to analyze and simultaneously segment lung and heart US images using a U-Net based architecture. One of the challenges with these approaches is that they can lead to a “multi-scale’’ problem. This is because every neural network model has its own receptive field scale, but organs in US images vary in size and scale. Therefore, a single scale may not accurately segment all organs. However, in a recent study, the mentioned problem has been addressed by their proposed multi-scale model with an attention mechanism by extracting multi-scale features from images with additive attention gate units for irrelevant feature elimination. Their dataset consisted of 312 US images of the fetal heart and lungs. The images, however, were acquired from a single source, which can lead to an overfitting problem and a relatively low number of images. Nevertheless, the simultaneous segmentation capability of this model has great potential because it allows a more holistic view of fetal anatomy to assess developmental anomalies. In addition, it can also allow for efficient single-pass processing of US images [98].

Another recent study aimed to predict 24 objects within the fetal heart in the four-chamber view using a Mask-RCNN architecture. Instead of using the whole ultrasound, the researchers employed the four standard fetal heart images as input data. These objects comprised the four standard shapes of fetal heart views, 17 heart chamber objects for each view, and three types of CHD: atrial septal defect (ASD), ventricular septal defect (VSD), and atrioventricular septal defect (AVSD). The model achieved a DICE of 89.70% and IoU of 79.97 [99]. However, it is worth noting that their DL-based approach was evaluated using a relatively small dataset of 1149 fetal heart images. Additionally, the study was conducted using data from a single center, which may limit the generalization of the results to other populations.

Xu Lu et al. proposed a novel approach to segmenting the apical four-chamber view in fetal echocardiography. Their method employs a cascaded CNN referred to as DW-Net [100]. Cascaded CNNs connect multiple CNNs sequentially to learn hierarchical visual features. Unlike GANs for generative modeling or ensembles that combine different models, cascaded CNNs break down difficult vision tasks into smaller problems that can be solved efficiently in a pipeline. As an advantage, they can scale to very deep networks. However, it can be resource-intensive to train each CNN individually, and errors may propagate across the entire network. The DW-Net model provided by Xu et al. comprises two sequential stages. The initial stage produces a preliminary segmentation map, while the subsequent refinement stage enhances the map’s accuracy. Their proposed approach enhances the reliability of identifying the defects by employing the DW-Net architecture with its dual-stage segmentation process. The cascaded neural network’s ability to generate refined segmentation maps ensures that subtle structural variations and anomalies within the fetal heart can be accurately determined. However, the dataset used for training and evaluation was still relatively small as it included 895 images from only healthy fetuses, and the apical four-chamber view was studied. In another study, Xu et al. developed a cascaded U-Net (CU-Net) that uses two branch supervisions to improve boundary clarity and prevent the vanishing gradient problem as the network gets deeper. It also benefits from connections between network layers to transfer useful information from shallow to deep layers for more precise segmentation. Additionally, their SSIM loss helps maintain fine structural details and produce clearer boundaries in the segmented images [101].

A recent study has introduced the multi-feature pyramid U-net (MFP-Unet), a novel deep-learning architecture for automated segmentation of the left ventricle (LV) in 2D echocardiography images [102]. MFP-Unet blends the U-Net and feature pyramid network (FPN) architectures to improve segmentation accuracy. Object recognition and image segmentation tasks are the focus of FPNs. FPNs enhance feature representation by creating a multi-scale hierarchy of feature maps through lateral connections and top-down pathways. This allows the network to collect both fine-grained and high-level contextual input, which ultimately enhances the network’s accuracy when detecting objects of varying sizes. This capability can be especially beneficial for medical images. For example, in identifying fetal heart defects in echocardiographic images, FPNs can assist by effectively detecting complex cardiac structures, ranging from subtle anomalies to the broader context of anatomical features. Their multi-scale approach is crucial in recognizing localized abnormalities and holistic heart structures. However, the FPN’s computational complexity and memory requirements may serve as limiting factors. Furthermore, the utilization of MobileNet, U-Net, and FPNs demonstrated a 14.54% increase in IoU compared to using only U-Net, when applied to the segmentation of a cardiac four-chamber image [103].

The proposed MFP-Unet model achieved an average DSC of 0.953 in a public dataset, outperforming other state-of-the-art models. The main innovation in this work is the combination of multi-scale feature pyramids with U-Net to enhance segmentation robustness and accuracy, along with “network symmetry and skip connections between the encoder-decoder paths” [102]. Skip connections are essential in neural networks because they help overcome training challenges, facilitate information flow, handle different scales of features, and promote faster convergence. Because of their small dataset of only 137 images, an augmentation method was used in this study. The researchers created 10 slightly different versions of the images by applying the elastic deformation method. Consequently, the augmentation of the image quantity by a factor of ten yielded a total of 1370 images. Each of these augmented images would be considered a new data point for training the neural network. By applying elastic deformation to the images, they introduced variations in the shape and appearance of the heart structures in the echocardiographic images. This augmentation technique helps the neural network learn to be more robust to different shapes and conditions it might encounter in real-world echocardiographic data. It is a common practice in deep learning to use data augmentation to artificially increase the size and diversity of training datasets when the original dataset is limited in size.

**Table 6:** Overview of key sections in fetal echocardiography. A summary of the purposes of different views of the fetal heart that are used in a standard fetal echocardiography procedure [104,105,106].

Section	Description	Purpose
Fetal Apical Four-Chamber Heart Section	View of the fetal heart from the apex, capturing all four chambers (left and right atria, left and right ventricles)	Assess size, structure, and function of each chamber individually and their alignment
Three-Vessel Catheter Section	Evaluates three major blood vessels in the fetus’s chest area: aorta, pulmonary artery, and superior vena cava	Assess size, position, and potential abnormalities of these vessels
Three-Vessel Trachea Section	Evaluates aorta, pulmonary artery, superior vena cava, and trachea simultaneously	Detect abnormalities involving both cardiovascular and respiratory systems
Right Ventricular Outflow Tract Section	Focuses on assessing the outflow tract of the right ventricle connecting to the pulmonary artery	Identify obstructions or malformations affecting blood flow from the right ventricle to the pulmonary artery
Left Ventricular Outflow Tract Section	Concentrates on evaluating the outflow tract of the left ventricle connecting to the aorta	Identify abnormalities or blockages hindering the flow of oxygenated blood from the left ventricle to the aorta

In a recent study protocol, Ungureanu et al. proposed a ML-based intelligent decision support system to analyze first-trimester fetal echocardiogram videos and help sonographers detect fetal cardiac anomalies. The system will then be validated on new US videos, with the primary outcome of improved anomaly detection in critical views of the heart by less experienced sonographers. Secondary outcomes assessed will be the optimization of clinical workflow and reduced discrepancies between evaluators. As a protocol, no results are presented since the study has yet to be conducted. However, this approach can be further investigated to help technicians in their diagnosis [105].

Yang et al. developed a DL-based classifier to identify ventricular septal defects. They obtained 1779 normal and abnormal fetal US cardiac images in the five standard views of the heart. They used five YOLOv5 networks as their primary model to classify images into “normal” and “abnormal”. According to the study, their model reached an overall accuracy rate of 90.67%. The performance of YOLOv5 was also compared to other mainstream recognition models, such as Fast RCNN and ResNet50, and Fast RCNN and MobileNetv2, and was found to be superior in terms of accuracy [107].

In addition to US image analysis, other approaches like cardiac QT signal processing have been used but require further research and assessment [108]. In another study, Dong et al. developed a DL framework comprising three CNN networks, namely, CNN, a deep-CNN, and an aggregated residual visual block net (ARVBNet), which is able to detect key anatomical structures on a plane. They aimed to build a fully automatic fetal heart US image quality control system. The model achieved the highest mean average precision (mAP) of 93.52% [109].

In another study, researchers examined the effectiveness of HeartAssist, an AI-based software designed to evaluate fetal heart health and identify any potential anomalies during the screening process. The study discovered that the quantity and percentage of images regarded as adequate visually by the expert or using HeartAssistTM were equivalent, with a percentage of more than 87% for all cardiac views examined. This indicates that using a program like HeartAssist to evaluate fetal cardiac problems during the second-trimester ultrasonographic screening for abnormalities has many potentials [110].

The mentioned studies can be used with other models to achieve a fully reliable automated system. For example, the work of Dong et al. [109], where they developed a CNN-based framework, could be used to automatically assess the quality of fetal US cardiac images before they are fed into the primary model for diagnosis. This helps ensure that only high-quality images are used for diagnosis, which can further improve the accuracy and reliability of the diagnosis.

#### 3.2.2. Head and Neck Anomalies

The development of the fetal brain is the most essential process that takes place during the 18–21 weeks of pregnancy. Any abnormalities in the fetal brain can have severe effects on various functionalities of the brain, such as cognitive function, motor skills, language development, cortical maturation, and learning capabilities [111,112]. Thus, a precise anomaly detection method is of the utmost importance. Currently, US is still the most commonly used method to initially examine the development of the fetal brain for any fetal anomalies during pregnancy. During the 18- to 21-week pregnancy period, US imaging is used to measure the cerebrum, midbrain, cerebellum, brainstem, and other regions of the brain as part of the screening for fetal abnormalities [113,114]. To detect fetal brain abnormalities, Sreelakshmy et al. developed a model (ReU-Net) based on U-Net and ResNet for the segmentation of fetuses’ cerebellum using 740 fetal brain US images [115].

The cerebellum is an essential part of the brain that plays a crucial role in motor control, coordination, and balance. The fetal cerebellum can be seen and distinguished from other parts of the brain in US images, which makes it relatively easy for technicians to examine it during scans and, consequently, for researchers to employ DL-based models for the segmentation of the obtained images. Moreover, ResNet is a popular model frequently used for medical image segmentation, and it offers to skip connections to address the vanishing gradient problem. More specifically, in deep networks, gradients that are used to guide the weight information update for layers can become smaller and smaller as they are multiplied at each layer, and they will eventually reach close to zero. This makes the network struggle to learn complex patterns from images, which is essential in medical image processing. Besides using ResNets, Sreelakshmy et al. also employed the Wiener filter, which reduces unwanted noises in most US images. As a result, their ReU-Net model achieved 94% and 91% for precision rate and DICE, respectively. Singh et al. also used the ResNet model in conjunction with U-Nets to automate the cerebellum segmentation procedure. However, in this study, by including residual blocks and using dilation convolution in the last two layers, they were able to improve cerebellar segmentation from noisy US images [116].

The subcortical volume development in a fetus is a crucial aspect to monitor during pregnancy. Hesse et al. constructed a CNN-based model for an automated segmentation of subcortical structures in 537 3D US images [117]. One important aspect of this research is the use of few-shot learning to train the CNN using relatively few manually annotated data (in this case, only nine). Few-shot learning is a machine learning paradigm characterized by the training of a model to perform various tasks using a very restricted amount of data. This quantity is often significantly smaller than what is typically required by conventional machine learning approaches. The basic goal of few-shot learning is to make models flexible and capable of doing tasks that would otherwise need extensive labeled data collection, which can be either time-consuming or expensive.

Cystic hygroma is an abnormal growth that frequently occurs in the fetal nuchal area, within the posterior triangle of the neck. This growth originates from a lymphatic system abnormality, which develops from jugular-lymphatic blockage in 1 in every 285 fetuses [118]. The diagnosis of cystic hygroma is made with an evaluation of the NT thickness. Studies have also shown the connection between cystic hygroma and chromosomal abnormalities in first-trimester screenings [119]. In this concern, a CNN model called DenseNet was trained by Walker et al. on a dataset that included 289 sagittal fetal US images (129 images were from cystic hygroma cases, and 160 were from normal NT controls) in order to diagnose cystic hygroma in the first-trimester US images. The model was used to classify images as either “normal” or “cystic hygroma”, with an overall accuracy of 93% [120]. Several studies have shown the advantages of DenseNet models over ResNet architectures in terms of achieving higher performance while requiring less computational power, along with parameter efficiency and enhanced feature reuse [121,122,123].

To perform US in order to look for abnormalities in the brains of prenatal fetuses, the standard planes of fetal brain are commonly used. However, fetal head plane detection is a subjective procedure, and consequently, prone to errors and mistakes by technicians. Recently, a study was conducted to automate fetal head plane detection by constructing a multi-task learning framework with regional CNNs (R-CNN). This MF R-CNN model was able to accurately locate the six fetal anatomical structures and perform a quality assessment for US images [124]. Similarly, Qu et al. proposed a method using differential CNNs for accurately identifying the six fetal brain standard planes. Unlike traditional CNNs that process each image independently, a differential CNN takes two input images and computes the element-wise difference between the corresponding pixels. This difference map, the differential image, is fed into the network for further processing. Large databases are necessary for researchers in this field, but they can also cause overfitting and other model limitations. The researchers used a dataset of images comprising 155 fetal images, which is a relatively small dataset. However, the researchers used several data augmentation methods, including rotation, flipping, and scaling, to increase the size of the training dataset to 30,000 images and to prevent the model from overfitting [125].

Lin et al. made a model that was trained on 1842 2D sagittal-view US images. It was made to find nine intracranial structures of the fetus, including the thalami, midbrain, palate, fourth ventricle, cisterna magna, NT, nasal tip, nasal skin, and nasal bone [126]. The study used both standard and non-standard sagittal-view ultrasound images. The researchers also used an external test set of 156 images from a different medical facility to assess the generalization, robustness, and real-world application of their fetus framework. This enabled them to evaluate how well the model performed beyond its initial training data, verifying that it could manage a wide range of clinical scenarios, patient demographics, and equipment variances. Unlike the Lin et al. model, which was also used for non-standard planes, the Xie et al. model was trained only on standard planes, which makes it prone to misjudgments if non-standard planes are presented. Additionally, this model only indicates that the cases are normal or abnormal, and lacks specificity regarding a clear and comprehensive diagnosis, which is necessary [127].

Based on the same dataset provided by Xie et al. [127], another study was conducted to develop a computer-aided framework for diagnosing fetal brain anomalies. Craniocerebral regions of fetal head images were first extracted using a DCNN with U-Nets and a VGG-Net network, and then classified into normal and abnormal categories. In small datasets, using VGG networks can lead to overfitting because of the large number of parameters available in these models. However, they used this model on a large dataset of US images and achieved an overall accuracy of 91.5%. In addition, the researchers implemented class activation mapping (CAM) to localize lesions and provide visual evidence for diagnosing abnormal cases, which can make them visually comprehensive for non-expert technicians. However, the IoU value of the predicted lesions was too low, and thus, more advanced object detection techniques are required for a more precise localization [128]. Furthermore, Sahli et al. proposed a SVM classifier to categorize fetal head US images into two categories: normal and abnormal. However, their database included images of fetuses with the same gestational age, which may limit the model’s generalization to diagnose fetal defects in images from different gestational ages [129]. In another recent study, researchers used 43,890 neurosonography images of normal and abnormal fetuses to build a DL-based model using the YOLOv3 architecture to find different patterns of fetal intracranial anomalies in standard planes and make a diagnosis for congenital CNS malformations. Their model is called the Prenatal Ultrasound Diagnosis Artificial Intelligence Conduct System (PAICS) and is capable of diagnosing ten different types of patterns. The micro-average AUC values for the PAICS range from approximately 0.898 to 0.981, indicating a high level of accuracy [130]. Real-time detection for tasks similar to this is essential for immediate diagnosis and decision making, especially if such models are eventually considered to be used in hospitals. In this case, Lin et al. used YOLOv3, which is known for its speed and efficiency in real-time object detection [131]. Unlike the previous study, which used CAM to localize lesions following their classification, YOLOv3 can simultaneously classify and localize anomalies in bounding boxes more accurately.

Other valuable information can be drawn from the segmentation of fetal head images in obstetrics for monitoring fetal growth [132]. This information is valuable for the assessment of fetal health. Everwijn et al. performed detailed neurosonography, including 3D volume acquisition, on fetuses with isolated CHD starting at 20 weeks of gestation. They used an algorithm to automatically evaluate the degree of fetal brain maturity and compare it between the CHD cases and the control group. The CHD cases were further categorized based on blood flow and oxygenation profiles according to the physiology of the defect. Subgroup analyses were then conducted. The results showed a significant delay in brain development in fetuses with CHD, especially those with transposition of the great arteries (TGA), which is a congenital heart defect where the two main arteries leaving the heart are switched (transposed), or intracardiac mixing, compared to the control group [133]. However, the study did not explain the reasons for these differences or whether they were only due to decreased oxygenated blood flow to the fetal brain. The authors have previously published another study on this matter and concluded that, compared to healthy control cases, fetuses with isolated congenital heart abnormalities had a slight delay in their cortical development [134].

Biometric parameters such as head circumference [135], biparietal diameter, and occipitofrontal diameter are commonly used in ultrasound examinations to assess fetal skull characteristics such as shape and size [59]. Zeng et al. developed a very lightweight DL-based model for a fast and accurate fetal head circumference measurement from two-dimensional US images [136]. Using the same dataset as the previous study, Wang et al.’s model achieved a DSC of 98.21% for the automatic measurement of fetal head circumference using a graph convolutional network (GCN), exceeding other state-of-the-art methods such as U-Net, V-Net, and Mask-RCNN [137]. Both of these studies used an augmentation method to increase the number of images. One important difference between the two studies was their efficiency in computation and memory demands. Lightweight DCNNs demand less computational power and memory compared to GCNs.

#### 3.2.3. Respiratory Diseases

The development and function of the lungs are crucial for the well-being and survival of fetuses. Malformations caused by underdevelopment or abnormalities inside the lung structure will lead to serious health issues and even death in newborns. For example, neonatal respiratory morbidity (NRM), such as respiratory distress syndrome or transient tachypnea of the newborn, is often seen when a fetus’ lungs are not fully developed, and it is still a major cause of morbidity and death [138]. Immature fetal lungs are closely linked to the respiratory complications experienced by newborns [139]. In addition, fetal lung lesions are estimated to manifest in around 1 in 15,000 live births, and are believed to originate from a range of abnormalities associated with fetal lung airway malformation [140]. In this case, the random undersampling with AdaBoost (RUSBoost) model was developed using extracted features from fetal lung images to predict NRM. However, locating regions of interest within the included images was manually performed, which is time-consuming and should be automated for use in clinics. This model was able to accurately predict NRM in fetal lung images. Small sample sizes and single-source datasets were also some of its limitations [141]. Du et al. conducted research comparing fetal lung texture using US-based radiomics technology in 548 pregnant women with gestational diabetes mellitus (GDM), pre-eclampsia (PE), and normal pregnancies at different gestational ages. Their model could differentiate fetal lung images associated with GDM/PE from normal cases [142].

There is a limited number of studies on using ML and DL models in lung malformations affecting fetuses. Owing to the importance of these conditions, more studies are needed to explore the potential of ML and DL in this area of medical image analysis.

#### 3.2.4. Chromosomal Abnormalities

Chromosomal disorders are frequently occurring genetic conditions that contribute to congenital disabilities. These disorders arise due to abnormalities in the structure or number of chromosomes in an individual’s cells, leading to significant health challenges and impairments present from birth. There are, however, various ways to detect them early on in the pregnancy. The ones that we are concerned with here are those evaluations that help us detect genetic disorders from US images. These include the following:

- NT measurement, which measures the thickness of the fluid-filled space at the back of the fetus’s neck.
- Detailed anomaly scan, a thorough US examination that checks for any structural abnormalities in fetuses.
- Fetal echocardiography, which focuses on evaluating the fetal heart structure and function to detect cardiac anomalies.
- Nasal bone (NB), whose absence is a valuable biomarker of Down syndrome in the first trimester of pregnancy.

In addition to the mentioned procedures, another technique that can be used to detect chromosomal disorders from US images is the measurement of fetal facial structure. Certain facial features can indicate the presence of certain genetic conditions [143]. For example, during a US screening, a technician will carefully examine the fetus’s facial structure for any abnormalities or distinctive features that may suggest a chromosomal disorder. For example, some common facial features of Down syndrome include a flat nasal bridge, upward-slanting eyes, and a small mouth. These features may be visible during a US and can raise the likelihood of a chromosomal disorder [144].

Tang et al. developed a two-stage ensemble learning model named Fgds-EL that uses CNN and RF models to train a model to diagnose genetic diseases based on the facial features of the fetuses. This study used 932 images (680 were labeled normal, and 252 were diagnosed with various genetic disorders). To detect anomalies, the researchers extracted key features from a fetal facial structure, such as the nasal bone, frontal bone, and jaw. These are specific locations where genetic disorders such as trisomy 21, 19, 13, and others can be identified. The CNN was trained to extract high-level features from the facial images, while the RF was used to classify the extracted features and make the final diagnosis. The proposed model achieved a sensitivity of 0.92 and a specificity of 0.97 in the test set [145].

**Figure 8:**
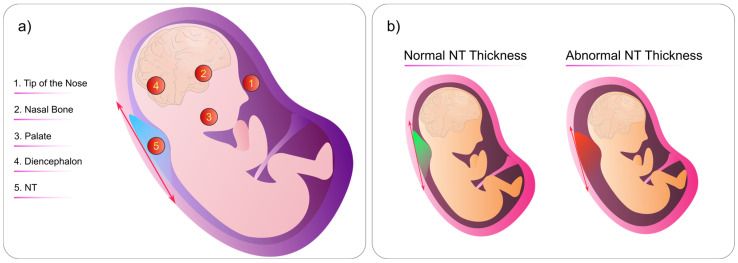
(**a**) Criteria for NT measurement. Specific locations on the fetus’s body where technicians can look for abnormalities. (**b**) Comparison of normal and abnormal fetal NT thickness. The abnormal NT thickness is significantly larger than the normal NT. This figure was generated by the authors using the Inkscape software version 1.3.

NT is the term used to describe the sonographic appearance of an accumulation of fluid under the skin of the fetus’s neck at around 11–13 weeks into the pregnancy (Figure 8b). Current research suggests that this measurement is crucial in assessing the risk of chromosomal abnormalities.

Currently, an NT measurement of 3.5 mm is considered an indication for invasive testing, often followed by chromosomal microarray analysis. In addition, fetal chromosomal abnormalities are not always accompanied by abnormal fetal karyotypes [146]. In this vein, one study found that when NT thickness is between the 95th centile and 2.5 mm, there is a potential existence of chromosomal abnormalities [25]. However, based on the quantitative results of another study, researchers concluded that the NT cut-off for invasive testing could be 3.0 mm instead of 3.5 mm [147].

Identifying NT abnormalities can be a difficult task, and researchers have found that the possibility of detecting fetal anomalies at the 11–13 week scan falls into the following categories [148]:

1. Always detectable
2. Never detectable
3. Sometimes detectable

In terms of NT measurement, there are specific locations on the fetal head where medical professionals look for abnormalities (Figure 8a):

1. Tip of the Nose
2. Nasal Bone
3. Palate
4. Diencephalon
5. Nuchal Translucency

By checking the mentioned locations, we can detect any abnormalities or variations in the thickness of the NT during the fetal US. Thus, any abnormalities in these areas can indicate potential genetic disorders or chromosomal abnormalities such as Down syndrome, various types of trisomy, and Turner syndrome [149]. Additionally, NT image segmentation using ML models has also shown to be effective for the early diagnosis of brain anomalies [150].

Down syndrome is the most frequent chromosomal abnormality and the most frequent cause of non-inherited mental retardation, characterized by a full or partial extra copy of chromosome 21. Children with Down syndrome often experience slower growth and have intellectual disabilities [151]. Thus, screening for trisomy 21 during the first trimester and early second trimester of pregnancy is crucial, so that mothers with affected fetuses can make informed decisions about their reproductive options as early as possible [152].

Most fetuses with trisomy 21 have a thicker NT and an absence of a nasal bone [153]. Babies born with trisomy 21 may have nasal bones that are underdeveloped or absent, resulting in a flat bridge. According to research, most fetuses with trisomy 21 lack a nasal bone. As a result, trisomy 21 is more likely in cases where the nasal bone is missing [154,155]. Another study found that the nasal bone-to-nasal tip length ratio might also be a potential marker for the diagnosis of trisomy 21 [156]. In a recently published paper, researchers employed an adaptive stochastic gradient descent algorithm to study the connection between NT thickness level and the potential existence of fetal anomalies. They collected 100 fetal US images to evaluate for anomalies. According to the authors, the accuracy of their model achieved 98.64% precision for classifying anomalies linked with NT thickness [157]. The previously mentioned Lin et al. model was also capable of NT identification [126].

Tekesin et al. demonstrated how valuable first-trimester US scanning can be performed by incorporating a detailed fetal anomaly scan into first-trimester screening algorithms, which is conducive to an improvement in the detection of trisomy 18 and 13, triploidies, and Turner syndrome [158,159]. Sun et al. developed a nomogram based on US images of fetuses with trisomy 21 in this context. Since nomograms are used in cases where multiple variables are available, they analyzed fetal profile images and identified facial markers and NT thickness. Based on the extracted markers, the LASSO (least absolute shrinkage and selection operator) method was used to make a prediction model for trisomy 21 screening in the first trimester of pregnancy. LASSO is a statistical method used for regression analysis. It adds a penalty term to the ordinary least squares method to shrink some of the coefficients to zero, effectively selecting the most critical variables and reducing model complexity. The resulting LASSO model achieved high accuracy, with AUC values of 0.983 and 0.979 in the training and validation sets, respectively [153]. The nomogram method for detecting Down syndrome using US images is simple, understandable, and does not need many data. It works well with limited resources and avoids overfitting by automatically selecting markers. Neural network models are good at finding complex patterns but need a lot of labeled data and computing power. This makes the nomogram a good choice, especially when data are limited or interpretability is essential.

Tang et al. developed a fully automated prenatal screening algorithm called Pgds-ResNet based on deep neural networks. Their model detected high-risk fetuses affected by various common genetic diseases, such as trisomy 21, 18, and 13, along with rare genetic diseases. Their dataset consisted of 845 normal images and 275 rare genetic disease images. Their feature extraction process indicated that the fetal nose, jaw, and forehead contained valuable diagnostic information [160]. However, their model was trained on a relatively small dataset from a single data center. Moreover, it was primarily designed for genetic abnormality screening rather than diagnosing specific conditions.

To detect trisomy 21, Zhang et al. constructed a CNN-based model using US images from 822 fetuses (548 were from normal fetuses and 274 were from fetuses diagnosed with trisomy 21). Their model was not only restricted to the NT thickness but successfully detected trisomy 21 based on images from the fetal head region with an accuracy of 89% in the validation set [161]. Nevertheless, one of the limitations of their model was that it was only trained to diagnose trisomy 21. There are cases where the fetus presents with more than one trisomy. Thus, developing a multi-task learning model for the simultaneous recognition of various types of trisomy is necessary [162,163].

## 4. Discussion

**Figure 9:**
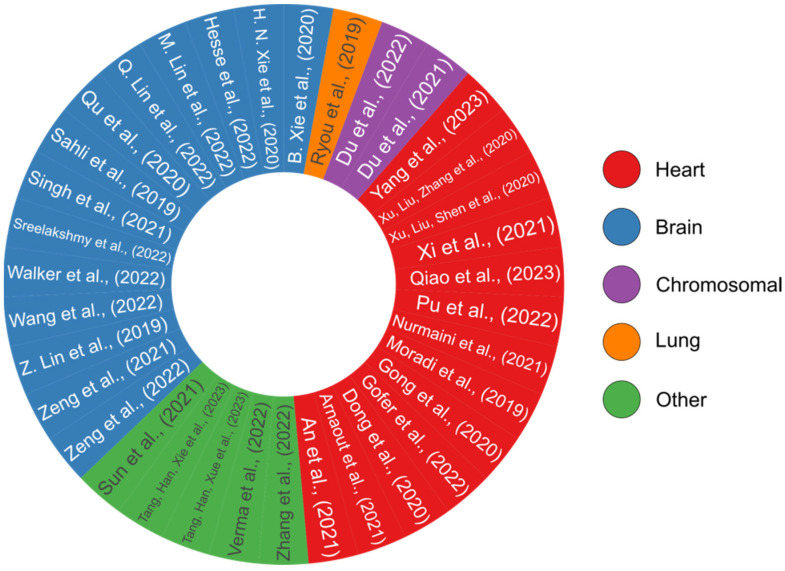
A pie chart overview of all the review papers that have presented DL-based models [58,95,96,97,98,99,100,101,102,103,107,109,115,116,117,120,124,125,126,127,128,129,130,135,136,137,141,142,145,150,153,157,160,161,169]. Each study’s color code reflects its relevance to a certain organ, including the heart, brain, lung, or analyzing for chromosomal abnormalities. This pie chart was generated by the authors using the R programming language.

Throughout this review, we examined some of the most recent methods for the detection of fetal anomalies such as heart defects, chromosomal abnormalities, head and neck malformations, and pulmonary disease (Figure 9). Along with anomaly detection, ML-based models for biometric measurement and locating the most effective standard planes were also reviewed (Table 7). While recent advancements hold promise, it is crucial to recognize the challenges that slow down the development of clinically applicable models in this domain.

**Evolution of Fetal Tissue:** One of the challenges in this field is the dynamic nature of fetal tissue, especially the brain, which constantly evolves during gestation. This inherent variability poses difficulties in training models to make precise and accurate diagnoses of abnormalities. Understanding the nature and patterns of this evolution is crucial in addressing this challenge effectively.

**Limited Labeled Datasets:** The small number of publicly available, high-quality labeled datasets and a reliance on single-source datasets contribute to the issue of overfitting in some models. To address this, various data augmentation techniques have been proposed, including elastic deformation and the utilization of advanced models such as GANs, diffusion models, variational autoencoders, and SVMNet [44,164]. Moreover, the application of few-shot learning techniques, as demonstrated in the Hesse et al. study [117], can be instrumental in enhancing the performance of models with limited data.

**Quality of Ultrasound Images:** Low-quality ultrasound images are a common issue in many datasets. To address this, quality assessment models, as highlighted by Zhang et al. [165], can be deployed to filter out subpar images, thus improving the overall dataset quality. The real-time detection of abnormalities is also vital for clinical adoption but remains an area that requires further exploration [166].

**Transfer Learning for Resource-Scarce Regions:** Countries with limited resources face additional challenges in accessing AI models. A potential solution lies in the application of transfer learning techniques [167,168]. These approaches involve amalgamating data from resource-rich regions with smaller samples from resource-scarce regions, offering a means to bridge the gap in healthcare accessibility.

**Overfitting and Network Depth:** Conventional deep neural networks encounter well-known issues like vanishing gradients and overfitting as their depth increases. These challenges can be mitigated through the incorporation of techniques such as regularization parameter tuning and the strategic use of skip connections, as exemplified by ResNets. The inclusion of skip connections not only alleviates the vanishing gradient problem but also streamlines the training process by reducing the need for large training sets.

**Multi-Scale Challenges in Image Analysis:** The inherent variability in organ sizes and scales within ultrasound images poses a significant hurdle. Traditional neural networks, with fixed receptive field sizes, struggle to capture relevant information across diverse dimensions. Researchers should consider the adoption of multi-scale architectures and techniques to ensure comprehensive feature extraction and the accurate analysis of organs of varying sizes within the same image.

**Table 7:** A summary of all reviewed studies on fetal anomaly detection. Each entry provides information about the employed methods, total number of images, key performance metrics, and application domain.

Method	Total Images	Metrics	Application	Refs
GACNN + DANomaly	3196	85.00%	Detection of heart defects	[95]
Ensemble of NN	107,823	AUC: 99%	Detection of heart defects	[96]
SOLOv2 + CAM	319	DICE: 74.70–81.99%	Segmentation of cardiac four-chamber view	[97]
U-Net + FCN	312	Heart: DICE: 90.2% IoU: 0.822 Lung: DICE: 87.00% IoU: 0.770	Segmentation of views of the lung and heart	[98]
Mask-RCNN	1149	DICE: 89.70% IoU: 79.97%	Detection of heart defects	[99]
Cascaded DW-Net	895	DICE: 82.7%	Segmentation of cardiac four-chamber view	[100]
U-Net + FPN	Original: 137 Augmented: 1370	Average DSC: 95.3%	Segmentation of cardiac four-chamber view	[102]
YOLOv5	1779	Overall accuracy: 90.67%	Detection of ventricular septal defects	[107]
CNN + D-CNN + ARVBNet	Original: 7032 Augmented: 12,542	MAP: 93.52%	Fetal heart image quality control system	[109]
U-Net + ResNet	740	DICE: 91%	Detection of fetal brain anomalies	[115]
U-Net + ResNet	734	DICE: 87.00%	Segmentation of the cerebellum	[116]
U-Net + ResNet	537	DICE: 85–90%	Segmentation of subcortical structures	[117]
R-CNN + Multi-task	1771	AUC: 98.89%	Quality assessment for fetal brain images	[124]
Differential-CNN	Original: 155 Augmented: 30,000	Accuracy: 92.93%	Identification of fetal brain standard planes	[125]
CNN	1842	AUC: 99.6%	Identification of intracranial structures	[126]
CNN	29,419	Segmentation: DICE: 94.1%; Classification: Overall accuracy: 96.3%	Detection of fetal brain anomalies	[127]
DCNN + U-Net + VGG	29,419	Overall accuracy: 91.5%	Detection of fetal brain anomalies	[128]
SVM Classifier	86	Accuracy: 87.10%	Classify fetal head US images	[129]
YOLOv3	43,890	AUC: 89.8–98.1%	Diagnose congenital CNS malformations	[130]
DenseNet	289	Overall accuracy: 93%	Detection of cystic hygroma	[120]
GCN	Original: 1334 Augmented: 11,324	DICE: 98.21%	Fetal head circumference measurement	[137]
Lightweight-DCNN	Original: 1334 Augmented: 10,898	DICE: 97.61%	Fetal head circumference measurement	[136]
RUSBoost	295	Accuracy: 81.18%	Detection of lung abnormalities: NRM	[141]
SVM Classifier	548	Accuracy (independent test set): 80.6–86.4%	Detection of lung abnormalities: GDM/PE	[142]
Ensemble Learning	932	Sensitivity: 97%	Detection of trisomy 21, 19, 13	[145]
Adaptive Stochastic Gradient Descent	100	Precision: 98.64%	Detection of chromosomal anomalies using NT thickness	[157]
Nomogram	622	AUC: 98.3–97.9%	Detection of trisomy 21	[153]
ResNet + VGG	1120	Sensitivity: Trisomy 21: 83%; Trisomy 18: 92%; Trisomy 13: 75%; Rare disorders: 96%	Detection of trisomy 21, 18, 13, and rare genetic disorders	[160]
CNN	822	Accuracy (validation set): 89%	Detection of trisomy 21	[161]
DAG V-Net (deeply supervised attention-gated)	1354	DICE: 97.93%	Fetal head circumference measurement	[135]
MobileNet + U-Net + FPN	677	IoU: 69.1%	Segmentation of cardiac four-chamber view	[103]
Cascaded U-Net	1712	DICE: 86.6%	Segmentation of cardiac four-chamber view	[101]
Feature Fusion GAN	1000	SSIM: 46.27%	Segmentation of cardiac four-chamber view	[58]
ImageJ/Fiji Software	80	NA	Detection of heart defects	[150]
FCN	65	Pixel mean accuracy: 89.4% ± 11.4	Whole fetus	[169]

## 5. Conclusions

In conclusion, the field of medical image analysis has made significant developments in recent years, with the advent of advanced DL models and data processing techniques that can significantly improve the quality of final models. Eventually, the developed models should be able to outperform sonographers and technicians in terms of accuracy and efficiency. These AI-driven models will not simply enhance the diagnostic process but also enable more personalized treatment plans based on individual patient data. Furthermore, the use of such models can reduce the workload of healthcare professionals, ultimately leading to a more streamlined healthcare system globally. However, several challenges still slow down progress in this area of research. As we mentioned, these challenges include the difficulty of training accurate models for diagnosing evolving fetal brain abnormalities, the lack of labeled ultrasound images for certain conditions, etc. Nevertheless, ongoing research and the advent of newer, more robust algorithms provide hope for the future.

## Figures and Tables

**Table 2:** Summary of advantages and disadvantages of popular DL-based models that are currently being used in a wide variety of tasks, including medical image analysis. Each model is different in its complexity, training time, and ability to deal with high-dimensional data, and has its own pros and cons for medical image analysis tasks.

Model	Advantages	Disadvantages	Refs.
Convolutional Neural Networks (CNNs)	Highly effective for medical image analysis. Automatically learn hierarchical features. Can handle various medical image modalities.	Require a large amount of labeled data. Computationally intensive and require GPUs. Susceptible to overfitting with small data.	[37,38,39]
Recurrent Neural Networks (RNNs)	Suitable for sequential medical data (e.g., time series). Can capture temporal dependencies (3D US videos). Useful for tasks such as electrocardiogram analysis.	Can suffer from vanishing gradient problem. Limited in handling very long sequences. Computationally expensive for deep networks.	[40]
Long Short-Term Memory (LSTM)	Mitigates vanishing gradient problem. Suitable for modeling temporal patterns. Effective for tasks like EEG signal analysis.	Complex architecture may lead to overfitting. Training may be slower than standard RNNs. Hyperparameter tuning can be challenging.	[41]
Gated Recurrent Unit (GRU)	Simpler than LSTM, easier to train. Suitable for sequential medical data. Requires less computation than LSTM.	May not capture long-term dependencies well. Limited in handling very long sequences.	[42]
Transformer	Effective for tasks like medical text analysis. Self-attention mechanism captures context. Can process variable-length sequences.	Initially designed for fixed-length inputs. May require a large amount of training data. Computationally intensive, needs GPUs.	[43]
Generative Adversarial Networks (GANs)	Can generate synthetic medical images for data augmentation. Useful for generating realistic medical images. Can be adapted for image-to-image translation tasks.	Training can be unstable and challenging. Mode collapse may lead to limited diversity. Requires careful tuning and monitoring.	[44]
Autoencoders	Useful for feature extraction in medical images. Can learn meaningful representations. Used for unsupervised learning and anomaly detection.	Need a clear objective for their use. Sensitive to noise in the input data. Architectural choices can impact performance.	[45]
U-Net	Designed for semantic segmentation tasks. Efficiently captures spatial information. Commonly used in medical image segmentation.	May require a large dataset for training. Prone to overfitting with limited data. May need architectural modifications for 3D data.	[46]
ResNet	Effective for very deep networks (residual connections). Addresses vanishing gradient problem. Achieves state-of-the-art results in image classification. Transfer learning-friendly architecture.	Increased model complexity. May require more data for training. Computationally intensive.	[47]

**Table 3:** Various standard planes for different fetal anatomical structures, as recommended by the International Society of Ultrasound in Obstetrics and Gynecology (ISUOG) guidelines. This criterion helps provide a systematic approach to ultrasound imaging in obstetrics by clearly defining the standard planes for key fetal anatomical structures. The purpose is to ensure a consistent and accurate visualization of these structures, irrespective of the ultrasound operator’s skill level. This approach aids in the early detection of fetal anomalies, helping with timely interventions if needed.

Standard Plane	Description
Fetal Abdomen (FASP)	Standard plane for extrapolating biometric measurements of the fetal abdomen.
Brain (FBSP)	Standard plane for extrapolating biometric measurements of the fetal brain.
Femur (FFESP)	Standard plane for extrapolating biometric measurements of the fetal femur.
Trans-Ventricular (FVSP)	Standard plane of brain imaging involving visualization through the ventricles.
Trans-Thalamic (FTSP)	Standard plane of brain imaging involving visualization through the thalamus.
Maternal Cervix	Standard plane for evaluating the maternal cervix.
Fetal Heart	Left Ventricular Outflow Tract (LVOT) Four-Chamber View (FCH) Right Ventricular Outflow Tract (RVOT) Three-Vessel Trachea (3 VT) Three-Vessel View (3 VV)
Fetal Trans-Cerebellum (FCSP)	Standard plane for imaging the fetal cerebellum.
Fetal Facial (FFSP)	Standard plane for imaging the fetal face. Includes axial (FFASP), coronal, and sagittal planes.
Lumbosacral Spine (FLVSP)	Standard plane for imaging the fetal lumbosacral spine.

**Table 4:** The table presents a comprehensive comparison of various neural network architectures commonly employed in the field of medical image processing. Each architecture is assessed across multiple characteristics, including architecture type, primary use case, network purpose, training approach, loss of function, and data augmentation methods that are commonly used along with them. These architectures have been employed for diverse applications such as image segmentation, generation, classification, and improved generalization. The table also highlights popular variants, references to relevant studies, and key attributes.

Ensemble of NNs	Cascaded CNN	CNN	GAN	U-Net	Characteristic
Combination of various networks	Feedforward	Feedforward	Generator-Discriminator	Encoder-Decoder	Architecture
Improved Generalization	Image Segmentation	Image Classification	Image Generation	Image Segmentation	Application
Improved Performance	Hierarchical Feature Extraction	Feature Extraction and Pattern Recognition	Image Generation and Enhancement	Segmentation and Feature Extraction	Network Purpose
Various (e.g., Bagging, Boosting)	Supervised	Supervised	Unsupervised	Supervised	Training Approach
Varies based on constituent nets	Cross-Entropy Loss	Cross-Entropy Loss	Adversarial Loss	DICE Coefficient Loss	Loss Function
N/A (Individual Networks)	N/A (Part of Cascaded CNN)	N/A (Part of GAN)	Generator Network	U-Net Architecture	Generator Network
N/A (Individual Networks)	N/A (Part of Cascaded CNN)	N/A (Part of GAN)	Discriminator Network	N/A (Part of GAN)	Discriminator Network
Combination of Features	Hierarchical Features	Hierarchical Features	N/A	Low-level and High-level Features	Feature Learning
Sometimes used	Occasionally used	Occasionally used	Rarely used	Commonly used	Data Augmentation
Varies based on constituent nets	Robust to noise	Robust to noise	Sensitive to noise	Can handle noise and incomplete data	Noise Handling
Improved Robustness, Accuracy	Hierarchical Feature Extraction	Hierarchical Feature Learning	Realistic Image Generation	Accurate Segmentation, Feature Localization	Advantages
Complexity and over-fitting	Complexity and over-fitting	Limited receptive field	Mode collapse	Requires sufficient training data	Challenges
Bagging, Boosting, Stacking	Cascade-CNN, Stacked CNN	VGG, ResNet, Inception	DCGAN, CycleGAN	U-Net++, U-Net 3+	Popular Variants
[70,71]	[72]	[42]	[73,74]	[46,75]	Refs

**Table 5:** Appropriateness criteria for fetal anomaly screening in second- and third-trimester pregnancies. These criteria can help ensure that any potential risks or complications are detected early on. They also allow for the possibility of intervention, if necessary, to ensure the health of both mother and baby.

Variant	Status
Variant 1	Initial second- and third-trimester fetal anomaly screening in low-risk pregnancy is appropriate using a transabdominal ultrasound (US) pregnant uterus scan.
Variant 2	Initial second and third-trimester fetal anomaly screening in high-risk pregnancy is appropriate using a transabdominal detailed US pregnant uterus scan. Controversy exists around MRI and standard US use.
Variant 3	Soft marker identification on US anatomy scans suggests a subsequent transabdominal detailed scan and follow-up US scans, chosen based on marker type, to manage patient care effectively.
Variant 4	Significant anomalies found on US screening lead to a transabdominal detailed US, MRI fetal without IV contrast, US echocardiography, and follow-up US scans for comprehensive patient care management.

## Data Availability

Not applicable.
